# Seroprevalence of *Borrelia burgdorferi *sensu lato and *Anaplasma phagocytophilum *in Danish horses

**DOI:** 10.1186/1751-0147-52-3

**Published:** 2010-01-18

**Authors:** Marie GB Hansen, Mette Christoffersen, Line R Thuesen, Morten R Petersen, Anders M Bojesen

**Affiliations:** 1 Department of Veterinary Disease Biology, Faculty of Life Sciences, University of Copenhagen, Stigbøjlen 4, DK-1870 Frederiksberg C, Denmark; 2 Department of Large Animal Sciences, Faculty of Life Sciences, University of Copenhagen, Dyrlægevej 68, DK-1870 Frederiksberg C, Denmark

## Abstract

**Background:**

*Borrelia burgdorferi *sensu lato and *Anaplasma phagocytophilum *are able to infect horses. However, the extend to which Danish horses are infected and seroconvert due to these two bacteria is unknown. The aim of the present study was to evaluate the seroprevalence of *B. burgdorferi *sensu lato and *A. phagocytophilum *in Danish horses.

**Methods:**

A total of 390 blood samples collected from all major regions of Denmark and with a geographical distribution corresponding to the density of the Danish horse population were analyzed. All samples were examined for the presence of antibodies against *B. burgdorferi *sensu lato and *A. phagocytophilum *by the use of the SNAP^®^4DX ^® ^ELISA test.

**Results:**

Overall, 29.0% of the horses were seropositive for *B. burgdorferi *sensu lato whereas 22.3% were seropositive for *A. phagocytophilum*.

**Conclusions:**

Antibodies against *B burgdorferi *sensu lato and *A. phagocytophilum *are commonly found among Danish horses thus showing that Danish horses are frequently infected by these organisms.

## Background

The vector-borne bacteria *Borrelia burgdorferi *sensu lato (*B. burgdorferi *s. l.) and *Anaplasma phagocytophilum *infect horses in those parts of the world where *Ixodes *spp. are present. It has been estimated that up to 30-40% of horses in an endemic area are seropositive for *B. burgdorferi *s. l. [1]. Most of these horses will remain asymptomatic, while 5-10% of them are likely of developing clinical signs [1-4]. Similar, a previous study on *A. phagocytophilum*, have indicated that up to 50% of seropositive horses in endemic areas undergo a subclinical infection [5]. It is assumed that clinical equine granulocytic anaplasmosis is an overseen condition in most of Europe, as most horses recover spontaneously and therefore do not attract the attention of clinicians [6]. In Denmark, *B. burgdorferi *s. l. and *A. phagocytophilum *are transferred by the tick *Ixodes ricinus*. A Danish study from 2005 revealed that the tick density varies substantially between different regions of Denmark with highest density on the island of Bornholm (0.5-1.0 ticks/min. flagging) followed by Zealand, Funen, Middle-Jutland and East-Jutland (0.25-0.5 ticks/min. flagging), South-Jutland (0.15-0.25 ticks/min. flagging), and West and North-Jutland (0-0.15 ticks/min. flagging) [7]. An apparent increase in the tick density from 1984 to 1998 [8] fits well with a positively correlated relationship between warmer winters and longer spring and autumn periods and the density of *I. ricinus *[9,10].

The seroprevalence of *B. burgdorferi *s. l. and *A. phagocytophilum *among horses in Denmark has to our knowledge never been evaluated. Recent European studies on the seroprevalence of *B. burgdorferi *s. l. shows a prevalence of 47.8% seropositive horses in Slovakia [11], 25.6% in Poland [12], 16.8% in Sweden [13], 16.1% in Germany [14] and 6.3% in Turkey [15]. The seroprevalence of *A. phagocytophilum *in Europe varies from 83.3% in Holland [6], 16.7% in Sweden [13], 11.3% in France [16], 8.1% in Italy [17,18] to 6.5% in Spain [19]. Furthermore, a Swedish study reported that 4.5% of the examined horses were seropositive for both *B. burgdorferi *s. l. and *A. phagocytophilum *[13]. In 2005, Danish researchers made a seroprevalence study on the distribution of *B. burgdorferi *s. l. and *A. phagocytophilum *antibodies in Danish deer. The overall seroprevalence was 36.6% for *B. burgdorferi *s. l. Significant regional differences were found when Jutland was compared with the islands (Funen, Zealand, Lolland, Falster and Bornholm) with a seroprevalence of 27.1% versus 46.7%, respectively. In the case of *A. phagocytophilum*, all tested deer in three districts of North-Jutland were antibody negative although the average seroprevalence was found to be 42.6% ranging from 39.6% in Jutland to 47.6% of the islands [7].

The aim of the present study was to evaluate the seroprevalence of *B. burgdorferi *s. l. and *A. phagocytophilum *in Danish horses.

## Materials and methods

### Sample size

The sample size was calculated using the following formulas:

where *n *is the sample size, *E *is the margin of error, *r *is the fraction of response that we are interested in and  is the critical value for the confidence level of *c*.

As the prevalence of horses seropositive for *B. burgdorferi *s. l. and *A. phagocytophilum *vary considerably in areas comparable to Denmark [11-14], calculations were based on a situation where the test results from each individual horse could have 50%-50% test outcome (*r = *50%). A difference of more than 5% (*E*) to the expected 50%-50% outcome with a confidence of 95% (*c*) should be detected. Thus, with an estimated population of 200,000 horses (*N*) in Denmark, a sample size including 384 individuals was proposed. As the test kits came as pre-packed batches, 390 individual blood samples were taken.

### Selection of horses

**Figure 1:**
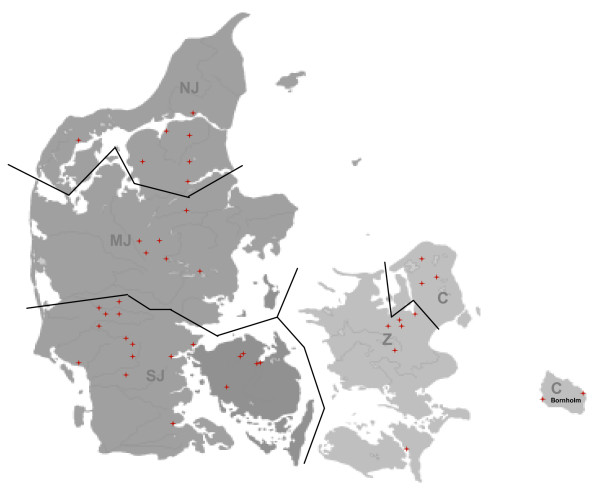
**Geographical location of sample sites (stars) within each of the five administrative regions of Denmark**. (NJ) Northern Jutland, (MJ) Mid Jutland, (SJ) Southern Jutland, (Z) Zeeland and (C) Capital region.

No formal randomized selection of horses was applied but an even representation of samples from all regions in Denmark was aimed at. Denmark is officially divided into five administrative regions including the Capital Region (C), Region Zealand (Z), Region South-Denmark (SD), Region Central-Jutland (CJ) and Region North-Jutland (NJ) (Figure 1). Assuming that the horse population is evenly distributed between the five regions, a sample size of 78 horses was used for each region. Within each region, horses were selected based on availability due to previous contact to individual horse owners, horse studs and clinical veterinary practices. Approximately one third of the samples were obtained from horses admitted to four equine veterinary clinics. The remaining samples were taken during visits to various size breeding farms, horse riding schools and farms. No horses with signs or a recent history of untreated infectious diseases were included.

### Serological evaluation of blood samples

A total of 390 blood samples were collected in EDTA blood collection tubes. All blood samples were analyzed immediately by a SNAP^®^4DX ^® ^ELISA test (IDEXX Laboratories, Taastrup, Denmark) according to the manufactures instructions.

### Putative risk factors

Every horse owner was asked to provide information with regard to putative risk factors including the breed, gender, age, use and housing, respectively. For each factor, 2-5 categories were established; "no answer" was included as a category. Gender included mare, stallion and gelding. Age was classified into groups of 1-4 years, 5-10 years, 11-20 years, and over 21 years. Housing facilities included pasture and pasture in combination with stabled housing, and access to pasture in the summer period was categorized as 1-3 months and >3 months. The horses were used for breeding, competitions/races, riding school and for leisure riding. Another risk factor was the use of the horse; it included the categories not used for riding, primarily used for leisure riding in the terrain or primarily used on a riding ground. Finally it was noted if the horse has been used for breeding.

### Statistical analyses

The ELISA results and their association with the putative risk factors were statistically analyzed using the PROC logistic procedure in SAS 9.1 (SAS Institute, Cary, NC, USA). A logit-transformation was used to estimate the relation between the outcome and the explanatory variables.

The model was given by:

Logit (*p_i_) = α + b(bacteria_i_) + c(region_i_)+ d(gender_i_) + e(age_i_) + f(housing_i_) + g(pasture in summer period_i_)+ h(breed_i_) + i(use_i_) + j(riding type_i_) + k(breeding_i_*)

Where *P*_*i *_is the dependent variable thefollowing outcomes was possible i) *B. burgdorferi *s. l. ii) *A. phagocytophilum*, iii) *B. burgdorferi *s. l. or *A. phagocytophilum *iv) both *B. burgdorferi *s. l. and *A. phagocytophilum*), *α *is the intercept, and *_i_*refers to the level of categories to the respective risk factor. The PROC logistic procedure tested two-way interactions between the different explanatory variables (risk factors). From the full model including all explanatory variables, a backward elimination was used to exclude non-significant variables. The parametric statistical Wald test was used to exclude the most non-significant variables until all variables in the model were significant.

To test the difference between the seroprevalence of *B. burgdorferi *s. l., *A. phagocytophilum *and the different regions, Chi square test or Fischer's exact test were used.

All statistical calculations were made with the software SAS 9.1. The level of significance was set to P < 0.05.

## Results

### Sampling

The sampled horses consisted of 222 mares, 135 geldings and 33 stallions. The breeds included Icelandic horse (182), Danish Warmblood (67), Jysk horse (11), Standardbred (11), Shetland Pony (10), Oldenburg (6), Friesian (6), Trakehner (5), Hanoverian (5), North Bagge (5), Connemara (4), Arabs (4) and other/mixed breeds (74). Horses aged 1 to 30 years (Mean = 9.2 years, SD ± 6.0) were included. All samples were obtained in the period from the 5^th ^of April to the 11^th ^of May 2009. The horses sampled originated from herds based in 42 individual postal codes (Figure 1). Of the horses tested through the participating veterinary clinics, 6-8 were under treatment and in the late recovery phase of an antibiotic treatment regimen, whereas the remaining horses were prophylactic cases including vaccination, teeth management, and the vast majority were subjected to assisted reproduction.

### Serology

The over-all seroprevalence of *B. burgdorferi *s. l. was 29.0% and 22.3% for *A. phagocytophilum*. The highest seroprevalence of 33.3% for *B. burgdorferi *s. l. was found in the Region of South-Denmark while only 24.4% of the samples from Region of North-Jutland were positive. The highest *A. phagocytophilum *seroprevalence (33.3%) was found in the Region of Zealand while the lowest (16.7%) was found in the Capital Region. Despite the seemingly high regional differences in seroprevalence for either organism, this could not be demonstrated statistically (*P *≥ 0.29). The regional seroprevalence distribution is summarized in Table 1.

**Table 1:** Seroprevalence of *Borrelia burgdorferi *sensu lato and *Anaplasma phagocytophilum *in 390 Danish horses.

Region	Borrelia-positive (%)	Anaplasma-positive (%)
Region North-Jutland	19 (24.4)	18 (23.1)
Region Central-Jutland	21 (26.9)	16 (20.5)
Region South-Denmark	26 (33.3)	14 (18.0)
Region Zealand	25 (32.1)	26 (33.3)
Capital Region	22 (28.2)	13 (16.7)
Denmark (Total)	113 (29.0)	87 (22.3)

A total of 78 horses were sampled from each region.

There was a considerable with-in region variation of the seroprevalences e.g. the Capital Region, which had an overall low seroprevalence for both *B. burgdorferi *s. l. and *A. phagocytophilum*. However, the seroprevalence for the island Bornholm, which belongs to the Capital Region was 60% for *B. burgdorferi *s. l. and 50% for *A. phagocytophilum*. Omitting the results from Bornholm, the Capital Region had a seroprevalence of 17.2% for *B. burgdorferi *s. l. and 5.2% for *A. phagocytophilum*. There was a significant lower seroprevalence of both *B. burgdorferi *s. l. (*P *= 0.017) and *Anaplasma phagocytophilum *(*P *< 0.001) in the Capital Region (Bornholm excluded) compared to Bornholm.

No significant association was found between any of the proposed risk factors and the occurrence of antibodies against *B. burgdorferi *s. l. (outcome (i). On the contrary, a significant correlation (*P *≤ 0.03) was found between the age of the horses and the presence of *A. phagocytophilum *antibodies (outcome ii). Horses aged 11 to 20 years had an odds ratio (OR) of 2.3 (with a 95% confidence interval of 1.2-4.6) for being seropositive for *A. phagocytophilum *whereas horses ≥ 21 years had an OR of 3.3 (with a 95% confidence interval 1.1-10.5) for being seropositive compared with horses aged 1-4 years, respectively. There was no significant association between other risk factors and presence of *A. phagocytophilum *antibodies. Eleven percent of the examined horses were seropositive for both *B. burgdorferi *s. l. and *A. phagocytophilum*. It appeared that the OR for horses seropositive for *B. burgdorferi *s. l. was 3.1 (with a 95% confidence interval of 1.9-5.0) when simultaneously being seropositive for *A. phagocytophilum *(outcome iii). Likewise, the OR for horses seropositive for *A. phagocytophilum *was 3.3 (with a confidence interval of 1.9-5.4) for simultaneously being seropositive for *B. burgdorferi *s. l. (outcome iii) Thus, there was a significant association between the occurrence of antibodies against *B. burgdorferi *s. l. and *A. phagocytophilum *(*P *≤ 0.0001) (outcome iiii) There was no significant association between the other factors investigated and the occurrence of a simultaneous infection with *B. burgdorferi *s. l. and *A. phagocytophilum*.

## Discussion

Comparing the seroprevalences of *B. burgdorferi *s. l. and *A. phagocytophilum *with results from neighboring European countries [insert refs], the seroprevalences found in this study are considerably higher. One important point explaining this difference could relate to sampling bias i.e. horses included in our study could have had a higher exposure risk to infected ticks than the general population. Although a formal randomized selection of horses was not applied, the considerable differences in geographic location, breed, age and use of the horses sampled is believed to provide a good approximation of the general horse population in Denmark. Furthermore, none of the horses included this study were selected because they exhibited or have had clinical signs of equine borreliosis or equine granulocytic anaplasmosis. On the contrary, previous studies have shown a steady increase in the ticks density, which has been attributed to climatic changes [8] and an increased infection rate among the ticks [21], which may prove to be more likely reasons for the higher prevalences observed. Whether the differences in the prevalences in Denmark versus Sweden and Germany [13,14] are due to an increase in the number of infected ticks since the latter investigations were performed, or whether the prevalence in Denmark in fact is higher will however remain unknown until data from prevalence studies with a comparable design and method from all countries in the region are performed. Differences in study design e.g. serological test method and statistical approach for the analysis of the results currently makes comparison of previous results difficult.

The highest density of ticks has previously been found on Bornholm, whereas a lower density was observed in the major Danish islands and in Jutland [7]. In addition to this, a Danish study of the seroprevalence of *B. burgdorferi *s. l. in deer showed that the risk of becoming infested with a tick positive for *B. burgdorferi *s. l. was greatest in the Capital Region followed by the Region of Zealand and the regions of Jutland, respectively. Interestingly, the risk of a deer being positive for *B. burgdorferi *s. l. antibodies was two times higher for Bornholm (part of the Capital Region) than for the island Zealand in general [22]. The present study shows that most of the horses seropositive for *B. burgdorferi *s. l. were found in the Region of South-Denmark followed by the Region of Zealand, the Capital Region, and Region of Central-Jutland and the Region of North-Jutland, thereby largely reflecting the tick density. In cases where the results did not match the deer study [ref], this may reflect differences as to the locality sampled within the regions compared. In accordance with the above, the study also shows that the prevalence of horses seropositive for *B. burgdorferi *s. l. on the island of Bornholm was two times higher than for Zealand, when the results for Bornholm was omitted in the overall result of the Capital Region and the Region of Zealand. We analyzed blood samples taken during spring, which likely influenced the number of seropositive individuals negatively as the likelihood of becoming seropositive later during the summer and autumn due to a longer exposure and risk of being infested by an infected tick [13].

The SNAP^®^4DX ^® ^test was developed for screening of *Dirofilaria immitis *antigen and antibodies to *A. phagocytophilum, B. burgdorferi *and *Ehrlichia canis *in canine serum, plasma or whole blood [23,24]. However, SNAP^®^4DX ^® ^has previously been evaluated and found useful to detect antibodies against *B. burgdorferi *s. l. and *A. phagocytophilum *in equine samples [23]. Compared to Western immunoblot, the SNAP^®^4DX ^® ^was found to have a sensitivity of 100% and a specificity of 95% for detection of antibodies against *B. burgdorferi *s. l. in equine samples. Detecting *A. phagocytophilum *antibodies in equines by the SNAP^® ^4DX ^® ^test comparison to the indirect immunofluorescence assay showed a sensitivity and a specificity of 100% [25]. Johnson *et al. *[ref] found the SNAP^®^4DX ^® ^test less sensitive (63%) yet 100% specific when comparing it to Western blotting and a C6-ELISA during a longer course of experimental infection [28]. By using ELISA in the form of a SNAP^®^4DX^® ^test it should be noted that this method detects both active and previous infections with measurable antibody levels [20]. From previous studies, the antibody levels in horses have been found detectable for up to 2 years [20,26]. In these, the clinically affected horses had the highest ELISA titers compared to horses with subclinical infections [1]. From studies with horses experimentally infected with *B. burgdorferi *s. l. and subsequently analyzed by ELISA, it appeared that even in cases where the horses had no clinical signs, antibodies were detected within 5-6 weeks. Antibody levels rose to a maximum over the following 3-4 months after which they remained static for at least 9 months [27]. However, another study has shown that individual horses, despite being positive by PCR and cell culture, did not remain seropositive by the SNAP^®^4DX^® ^test over a period of 9 months [28]. The above indicates that the antibody level may be detectable by some ELISA tests at least 9 months post-infection with *B. burgdorferi *s. l. but presumably for a shorter period using the SNAP^®^4DX^® ^test. Experimental infections in horses inoculated intravenously with *A. phagocytophilum *resulted in seroconversion 6-8 days post inoculation and achieved a maximum ELISA titer at day 10-30. The antibodies remained detectable for up to 5 months [28]. It is well established that horses acquiring a natural infection with *A. phagocytophilum *mount a slower antibody response, likely since the burden of infection typically is lower and delivered over a prolonged period [29]. Despite a longer incubation period, prior seroconversion from a natural infection versus the intravenously administered infections, the antibody levels were similar after 30 days [30]. Therefore, it is reasonable to assume that an ELISA may have a positive outcome for up to 5 months post-infection with *A. phagocytophilum. *The results found in this study therefore likely reflect the proportion of horses that have been infected with *B. burgdorferi *s. l. within the last 9 months or *A. phagocytophilum *within the last 5 months from the time of sampling and not only horses with an active infection at the time of blood sampling.

## Conclusions

The present study demonstrates that antibodies against *B. burgdorferi *s. l. and *A. phagocytophilum *can be commonly found in Danish horses. The findings warrant further attention to these infections in horses particularly with regard to improved means for detection of active infections, which may contribute to a better general understanding of these diseases and their impact on horse behavior and welfare.

## Competing interests

The authors declare that they have no competing interests.

## Authors' contributions

MGBH participated in the design of the study and carried out the sampling and ELISA tests, contributed to the statistical analysis and drafted the manuscript. MC and LRT performed the statistical analysis. MRP participated in the design of the study and helped to draft the manuscript. AMB participated in the design of the study, coordinated the activities and helped to draft the manuscript. All authors read and approved the final manuscript.

## References

[B1] Manion TB Bushmich SL Mittel L Laurendeau M Werner H Reilly M Manion TB, Bushmich SL Lyme disease in horses: Serological and antigen testing differences44th Proceedings of the Annual Convention of the AAEP: 6-9 December 1998; Baltimore 1998 144 145

[B2] Bushmich SL Lyme borreliosis in domestic animals J Spiro Tick Dis 1994 1 24 28

[B3] Magnarelli LA Anderson JF Shaw E Post JE Palka FC Borreliosis in equids in northeastern United States Am J Vet Res 1988 49 359 362 3282461

[B4] Myhre G Orcutt R Lyme disease: Insight into prevalence diagnosis and treatment J Equi Vet Sci 2008 28 390 391 10.1016/j.jevs.2008.05.002

[B5] Madigan JE Hietala S DeRock E Seroepidemiologic survey of antibodies to *Ehrlichia equi *in horses of northern California J Am Vet Med Assoc 1990 196 1962 1964 2195000

[B6] Butler CM Nijhof AM Jongejan F Kolk JH van der *Anaplasma phagocytophilum *infection in horses in the Netherlands Vet Rec 2008 162 216 217 18281631 10.1136/vr.162.7.216

[B7] Skarphédinsson S Jensen PM Kristiansen K Survey of tickborne infections in Denmark Emerg Infect Dis 2005 11 1055 1061 16022780 10.3201/eid1107.041265 PMC3371797

[B8] Jensen PM Frandsen F Temporal risk assesment for Lyme borreliosis in Denmark Scand J Infect Dis 2009 32 539 544 10.1080/003655400458848 11055661

[B9] Bennet L Halling A Berglund J Increased incidence of Lyme borreliosis in southern Sweden following mild winters and during warm humid summers Euro J Clin Microbiol Infect Dis 2006 25 426 432 10.1007/s10096-006-0167-2 16810531

[B10] Lindgren E Tälleklint L Polfeldt T Impact of climatic change on the northern latitude limit and population density of the disease-transmitting European tick *Ixodes ricinus* Environ Health Perspect 2000 108 119 123 10.2307/3454509 10656851 PMC1637900

[B11] Stefancíková A Derdáková M Škardová I Szestáková E Cisláková L Kovácová D Stanko M Petko B Prevalence of antibodies to *Borrelia burgdorferi *in horses of east Slovakia Vet Med Czech 2000 45 227 231 [in Czech]

[B12] Stefanciková A Adaszek L Petko B Winiarczyk S Dudinák V Serological evidence of *Borrelia burgdorferi *sensu lato in horses and cattle from Poland and diagnostic problems of Lyme borreliosis Ann Agric Environ Med 2008 15 37 43 18581977

[B13] Egenvall A Franzén P Gunnarsson A Engvall EO Vågsholm I Wikström UB Artursson K Cross-sectional study of the seroprevalence to *Borrelia burgdorferi *sensu lato and granulocytic *Ehrlichia *spp. and demographic, clinical and tick-exposure factors in Swedish horses Prev Vet Med 2001 49 191 208 10.1016/S0167-5877(01)00187-8 11311953

[B14] Käsbohrer A Schönberg A Serologic studies of the occurrence of *Borrelia burgdorferi *in domestic animals in Berlin (West) Berl Münch Tierärztl Wochenschr 1990 103 374 378 [in German] 2268252

[B15] Bhide M Yilmaz Z Golcu E Torun S Mikula I Seroprevalence of anti-*Borrelia burgdorferi *antibodies in dogs and horses in Turkey Ann Agric Environ Med 2008 15 85 90 18581984

[B16] Leblond A Pradier S Pitel PH Fortier G Boireau P Chadoeuf J Sabatier P An epidemiological survey of equine anaplasmosis (*Anaplasma phagocytophilum*) in southern France Rev Sci Tech 2005 24 899 908 [in French] 16642760

[B17] Passamonti F Fabrizia V Katia C Stefano C Giacomo C Luisa MM Daniela PF Andrea VS Mauro C *Anaplasma phagocytophilum *in horses and ticks: A preliminary survey of central Italy Comp Immunol Microbiol Infect Dis 2010 33 73 83 10.1016/j.cimid.2008.08.002 18805584

[B18] Torina A Vicente J Alongi A Scimeca S Turlá R Nicosia S DiMarco V Caracappa S de la Fuente J Observed prevalence of tick-borne pathogens in domestic animals in Sicily, Italy during 2003-2005 Zoonoses Public Health 2007 54 8 15 10.1111/j.1863-2378.2007.00989.x 17359441

[B19] Amusategui I Sainz A Tesouro MA Serological evaluation of *Anaplasma phagocytophilum *infection in livestock in northwestern Spain Ann N Y Acad Sci 2006 1078 487 490 10.1196/annals.1374.091 17114760

[B20] Dessau RB Bangsborg JM Ejlertsen T Hansen K Lebech A Østergaard C Laboratory diagnostics of infections caused by *Borrelia burgdorferi* Ugeskr Læger 2006 168 2805 2807 [in Danish] 16942701

[B21] Vennestrøm J Egholm H Jensen PM Occurrence of multiple infections with different *Borrelia burgdorferi *genospecies in Danish *Ixodes ricinus *nymphs Parasitol Int 2008 57 32 37 10.1016/j.parint.2007.07.004 17804280

[B22] Jensen PM Hansen H Frandsen F Spartial risk assesment for Lyme borreliosis in Denmark Scand J Infect Dis 2000 32 545 550 10.1080/003655400458857 11055662

[B23] IDEXX Laboratories Package Insert 2009 http://www.idexx.com/animalhealth/testkits/4dx/060505707.pdf

[B24] IDEXX Laboratories SNAP 4Dx Test 2009 http://www.idexx.com/animalhealth/testkits/4dx/

[B25] Chandrashekar R Daniluk D Moffitt S Williams J Serologic diagnosis of equine borreliosis: Evaluation of an in-clinic enzyme-linked immunosorbent assay (SNAP 4Dx) Intern J Appl Res Vet Med 2008 6 145 150

[B26] Reed SM Bayly WM Sellon DC Equine Internal Medicine 2004 1 Louis: Saunders

[B27] Chang YF Novosol V McDonough SP Chang C-F Jacobson RH Divers T Quimby FW Shin S Lein DH Experimental infection of ponies with *Borrelia burgdorferi *by exposure to Ixodid ticks Vet Pathol 2000 37 68 76 10.1354/vp.37-1-68 10643983

[B28] Johnson AL Divers TJ Chang Y-F Validation of an in-clinic enzyme-linked immunosorbent assay kit for diagnosis of *Borrelia burgdorferi *infection in horses J Vet Diagn Invest 2008 20 321 324 18460618 10.1177/104063870802000309

[B29] Pusterla N Lutz H Braun U Experimental infection of four horses with *Ehrlichia phagocytophila* Vet Rec 1998 143 303 305 9789347 10.1136/vr.143.11.303

[B30] Franzén P Aspan A Egenvall A Gunnarsson A Åberg L Pringle J Acute clinical, hematologic, serologic and polymerase chain reaction findings in horses experimentally infected with a European strain of *Anaplasma phagocytophilum* J Vet Intern Med 2005 19 232 239 10.1892/0891-6640(2005)19<232:ACHSAP>2.0.CO;2 15822569

