# Seroprevalence of Borrelia burgdorferi sensu lato and Anaplasma phagocytophilum in Danish horses

**DOI:** 10.1186/1751-0147-52-49

**Published:** 2010-08-18

**Authors:** Marie GB Hansen, Mette Christoffersen, Line R Thuesen, Morten R Petersen, Anders M Bojesen

**Affiliations:** 1 Department of Veterinary Disease Biology, Faculty of Life Sciences, University of Copenhagen, Stigbøjlen 4, DK-1870 Frederiksberg C, Denmark; 2 Department of Large Animal Sciences, Faculty of Life Sciences, University of Copenhagen, Dyrlægevej 68, DK-1870 Frederiksberg C, Denmark

## Correction

After publication of this work [1] it has come to our attention that one of the references used in the article [2] has been incorrectly cited. The sentence "The seroprevalence of *A. phagocytophilum *in Europe varies from 83.3% in Holland" in the second paragraph of the Background section of the article is incorrectly written and should state "The PCR detection of *A. phagocytophilum *in Europe varies from 9.8% in Holland" instead. The authors apologise for any confusion caused.
